# A content analysis of the characteristics of the popular mental health podcasts

**DOI:** 10.1093/her/cyag027

**Published:** 2026-09-10

**Authors:** Romy RW, Regina A A Alcántara, Malee Bedolla

**Affiliations:** Loyola Marymount University, Foley Hall 327, Loyola Marymount University, Los Angeles, 90045, California; Loyola Marymount University, Foley Hall 327, Loyola Marymount University, Los Angeles, 90045, California; Loyola Marymount University, Foley Hall 327, Loyola Marymount University, Los Angeles, 90045, California

**Keywords:** Podcasts, mental health, PodCred Framework

## Abstract

Mental health challenges continue to rise in the United States, yet access to treatment remains limited. Podcasts have become an important channel for disseminating mental health information and improving mental health. Despite the proliferation of mental health podcasts, little is known about the characteristics that make certain podcasts especially popular. To address this gap, the present study conducted a comprehensive content analysis of the top 100 mental health podcasts listed in the Apple Podcasts app and guided by the PodCred Framework. Three trained coders analyzed 100 mental health podcasts. Findings revealed consistent patterns among top-ranked podcasts. Most of them maintained a strong topical focus, consistent structure, and conversational delivery style, with episodes averaging 30–60 minutes. Podcasters frequently shared personal experiences, displayed emotional expressiveness, and demonstrated professional credibility through credentials and affiliations. Content typically combined recommendations, factual information, and personal discussion, supported by high production quality and predictable release schedules. Overall, the results extend the PodCred Framework to the mental health domain and highlight key features that appear to attract and sustain listeners. These insights offer practical guidance for podcast creators and contribute to emerging scholarship on digital mental health communication.

## Introduction

The prevalence of mental health issues among American adults is staggering, with more than 61.5million individuals experiencing mental health challenges and approximately 14.6 million classified as having a serious mental illness [1]. Additionally, analytics have shown that in the past decade, there have been substantial increases of symptoms consistent with major depression, serious psychological distress, and suicidal thoughts [2]. Despite the growing prevalence of mental health concerns, access to treatment remains limited. Among adults with any mental illness (AMI), roughly half did not receive mental health treatment in the past year [1]. This surge in unmet need has placed considerable strain on mental health providers, with many psychologists reporting an inability to keep up with demand and increased experiences of burnout [3]. Amid this escalating demand, promoting mental health literacy and awareness grows increasingly vital. Mental health literacy encompasses individuals’ ability to access, understand, and navigate the mental health care system. Higher levels of literacy are associated with improved recognition of symptoms, engagement in self-help behaviors, and timely treatment seeking. Conversely, limited awareness fosters stigma, misinformation, and avoidance of help-seeking [4]. Research further indicates that mental health literacy is unevenly distributed, with marginalized, lower socioeconomic, older, and less educated populations exhibiting lower fluency and more stigmatized discourse surrounding mental health [5].

Social media has proven to be an effective method of distributing information on the scale that this mental health epidemic demands, especially among communities which have historically had less access. With over 54% of Americans using social networking sites, the ease and reach that media campaigns can have is astronomical. Social media also provides the sense of interpersonal connection essential for health communication interventions, allowing people to be reached on the emotional and rational levels needed to catalyze a change [6]. Studies have shown that following a campaign exposure, both awareness and interest was reported by participants, with reports of reduced stigma and an increase in abilities to cope with mental health issues [7]. Alongside social media, podcasting, a form of new digital media that extends audio storytelling beyond broadcast radio [8], has experienced a meteoric rise in listenership and popularity in the last decade. Globally, the podcast listener base now exceeds 500 million people [9]. The popularity boom can be attributed to the convenient form of entertainment podcasts offer, away from a screen or a book. They are seen as a productive use of time and can be listened to in conjunction with other mindless tasks [10]. The medium has a unique ability to distribute a steady stream of information and entertainment while also keeping the personal connection essential for active communication to the listener. Due to this, it has a capacity for raising awareness, empowerment, providing community, and causing change [11]. While prior research highlights the impact of mental health podcasts in raising awareness and knowledge [5, 10], little is known about the factors that make certain mental health podcasts more popular than others. Therefore, the current project aims to conduct a comprehensive content analysis of existing mental health podcasts, with a particular focus on identifying the characteristics of these popular podcast episodes.

## Literature review

### Mental health podcasts

Podcasts have been used in mental health contexts, offering guidance, meditations, and psychoeducation, has proliferated [10], as audiences actively seek mental health-related content. Evidence suggests that mental health podcasts have been used to promote mental well-being by providing accessible, relatable content that can enhance knowledge, reduce stigma, promote self-awareness, and delivers mindfulness practices, underscoring their potential as psychoeducational tools that integrate psychological and pedagogical principles to foster personal growth [5, 12, 13].

While the high application potential of mental health podcasts has spawned some research, it is still a relatively newer field. Previous studies found that the most popular podcasts among listeners were interview style, with guests detailing lived experiences. This format facilitated a parasocial relationship between listeners and the podcaster, supporting literature around the connection which develops through the intimacy of podcast storytelling [14]. Another study compiled expertise from qualified individuals to find elements which created an engaging podcast, designed to reduce stigma. The results produced a hypothetical podcast of shorter length, semistructured, with a mix of casual and educational content, and celebrity and everyday guests talking about lived experiences, though the podcast has yet to be produced and tested [10]. Though these studies indicate the positive relationship between mental health podcasts and the mental health attitudes and practices of listeners, little research has been done to examine the characteristics of the most popular mental health podcasts.

### Parasocial relationship theory

Parasocial relationship theory, originally articulated by Horton and Wohl [15], describes the one-sided yet psychologically meaningful sense of intimacy that media users develop with on-screen and on-air personae. Although the construct was initially formulated to explain audience attachment to television hosts, subsequent research has extended it to podcasting [16]. Three antecedents of parasocial bonding have been consistently identified across these contexts: persona self-disclosure, direct address to the audience, and emotional expressiveness [16–18]. In the podcasting context, these antecedents carry particular weight. The intimacy of the audio medium, combined with the regularity of episodic release, positions podcast hosts as familiar and trusted voices in listeners’ daily routines, often encountered during private, low-distraction activities such as commuting, exercising, or preparing for sleep [16]. These conditions make the medium well suited to the formation of parasocial bonds, even without any reciprocal interaction.

For mental health podcasts specifically, parasocial bonding takes on particular theoretical and practical weight. Listeners who are navigating psychological distress, stigma, or uncertainty about seeking help may be especially drawn to hosts who disclose personal vulnerabilities, speak directly and empathetically to the audience, and maintain emotional consistency across episodes. These communicative behaviors satisfy the antecedent conditions of parasocial bonding and, at the same time, reflect rapport, validation, and normalization. This may help explain why audiences return repeatedly to programs built around a single, recurring host, and why personal narrative is among the most common content strategies in popular mental health podcasting. The present study applies parasocial relationship theory to examine how self-disclosure, affective delivery, and direct listener address shape the communicative character of top-ranked mental health podcasts, and to better understand why this content sustains the kind of audience engagement it does.

### Uses and gratifications theory

Uses and Gratifications Theory (U&G; [19, 20]) shifts the analytic focus from what media do to audiences toward what audiences actively do with media. Rather than treating listeners as passive recipients of media effects, U&G frames them as goal-directed agents who select, attend to, and interpret media content in ways that fulfill specific psychological and social needs. These needs have been broadly organized into five categories: cognitive gratifications (information and knowledge acquisition), affective gratifications (emotional experience and regulation), personal integrative gratifications (self-understanding and identity affirmation), social integrative gratifications (connection and belonging), and tension-release gratifications (entertainment and escapism) [19, 20]. Within podcasting specifically, audience research has identified a gratification profile that includes convenience and portability, in-depth learning, habitual companionship, and parasocial emotional support [21–23], distinguishing the medium from broadcast radio and television and reflecting its on-demand, intimate character.

Mental health podcasting offers a particularly rich context for U&G analysis because the gratifications listeners seek are not merely hedonic but purposive and need-driven. Audiences facing barriers to formal mental health care, including cost, stigma, waitlists, and geographic access, may turn to mental health podcasts to meet specific informational and social needs that go unmet elsewhere, such as symptom recognition and normalization, psychoeducation, stigma management, emotional validation, and a sense of connection with others in similar circumstances [5, 10]. These are not incidental gratifications but deliberate, goal-directed ones, pursued by listeners who bring considerable motivation and selectivity to their media choices. This positions the mental health podcast listener among the more purposive media users in the U&G literature, making gratification-based explanations of podcast selection and sustained engagement especially applicable here.

### PodCred framework

The PodCred Framework was originally validated on a heterogeneous corpus of general-interest podcasts drawn from commercial podcast directories, with assessor judgments as the criterion against which surface-level credibility indicators were tested [24]. The indicators feed into four categories: content, podcasters, podcast context, and technical execution. The content category deals with the consistency and quality of the intellectual podcast content. It ensures that the material fulfills the informational needs of its users, including factors such as topical focus, type composition, source of content, and factual *versus* testimonial information. Podcasters identify the importance of credibility, measuring the expertise and trustworthiness of the podcaster. Here, style and quality of speech are examined, as well the podcasters background and history with the material. Podcast context, the third category, deals with the associated web of information sources and social players a podcast uses to build its repertoire. The indicators identify how tightly integrated it is with users, as well as the presence of possible biased information through ads, sponsors, and stores. Finally, Technical Execution determines the quality, time, and effort put into the podcast, and including indicators surrounding production, packaging, and distribution. The framework is unique in that the indicators are not specified as positive or negative in their influence on podcast attractiveness, nor is it specified that all are required for a podcast to be deemed successful in all settings.

PodCred Framework has been cited in multiple studies, the majority of them concerning podcast consumption patterns. One study used the Framework as it examined the challenges and future directions for podcasts. The categories identified by PodCred highlighted podcasts’ uniqueness compared to other media and identified a need for podcast-oriented search and recommendation engines [25]. Another study acknowledged the work of PodCred Framework as one of the only prior studies in user podcast preferences as it sought to recommend podcasts to users based on their Spotify song preferences. The results indicated a higher number of minutes listened and podcasts followed with no prior knowledge of a user’s podcast preferences [26]. A third study investigated user goals and retrieval technology, finding that while people listen and actively search for specific topics, there is no awareness of a content-based search for podcasts, though audiences responded positively to this idea [27]. The usage of PodCred Framework has mainly been examined as a reference for the potential of podcasts, and virtually no studies have been found which have actively tested the PodCred Framework against a certain niche. There is a noticeable gap of literature covering its applications in the rapidly proliferating mental health arena, especially pertaining to podcasts in that sector.

Three limitations of the original validation are relevant to the present application. First, while PodCred systematically identifies the surface-level observable features that characterize podcasts listeners prefer, it only documents what features are present but does not account for why those features attract and sustain audience engagement. U&G fills this explanatory gap by supplying the motivational logic underlying listener selection. Specifically, each of PodCred’s four dimensions can be mapped onto the distinct gratifications that mental health podcast listeners are likely to seek. The Content dimension, which captures topical focus, source citation, and structural consistency, corresponds to cognitive gratifications, satisfying listeners’ needs for credible, organized, and personally relevant health information. The Podcaster dimension, encompassing credentials, self-disclosure, affective delivery, and direct audience address, corresponds to affective and social integrative gratifications by providing emotional validation, relatable modeling, and a sense of interpersonal connection. The Podcast Context dimension, which includes listener interactivity, community forums, and references to current events, addresses social integrative gratifications by positioning the podcast as a shared communal space rather than an isolated consumption experience. Finally, the Technical Execution dimension, encompassing production quality, reliable distribution, and consistent release schedules, satisfies convenience and habit-formation gratifications by enabling seamless, routine-embedded listening. This dimension-by-dimension correspondence between PodCred’s listener-compatibility logic and U&G’s gratification focus reflects the underlying premise that audiences select podcasts whose observable features most efficiently align with their motivations. Second, the framework was developed for general podcast recommendation and retrieval rather than for any particular content domain, the indicators were intentionally domain-general, leaving open the question of which cues carry the most weight within specialized genres. Third, because the framework treats indicators as descriptive rather than evaluative, it does not specify which combinations of cues are theoretically expected to predict popularity.

Despite these limitations, PodCred is theoretically appropriate for mental health podcasting for two reasons. First, mental health content places unusually high demands on perceived source credibility, and PodCred’s explicit focus on credentials, affiliation, and articulation maps directly onto credibility cues that listeners are likely to seek. Second, the framework’s attention to consistency and structural regularity aligns with the parasocial mechanisms outlined above, which depend on repeated, predictable contact [16].

The current study implements the PodCred Framework in the context of mental health podcasts. The overwhelming number of mental health podcasts provides an opportune opening to apply the PodCred Framework, as it was formulated to apply to specific niches in larger content bases, and will help users navigate the ever-growing database. The variety and distribution of different mental health topics, hosts, and formats allow our study to examine and differentiate podcasts based on an exacting checklist vetted by the framework. The PodCred Framework’s use of surface level indicators sorted into categories which gratify different segments of user’s needs allows the podcasts to be analyzed on a level which combines both theoretical and practical thinking, redefining our understanding of user motivation and needs in respect to the mental health podosphere. Thus, we ask the following research questions:

*RQs: What content characteristics (RQ1), podcaster characteristics (RQ2), podcast context features (RQ3), and technical execution elements (RQ4) are evident in top-ranked mental health podcasts?*

## Materials and methods

### Data collection

All podcasts were sourced exclusively from Apple’s Podcast application, as it offers a dedicated ‘Mental Health’ category that facilitates systematic and comprehensive sampling. In contrast, other platforms such as Spotify rely primarily on keyword-based search functions (*e.g.* ‘mental health’), which may limit the results by excluding relevant podcasts that do not explicitly include those terms in their titles or descriptions. We selected the top 100 channels listed under the Mental Health category in the Apple Podcasts app. This ranking reflects Apple’s proprietary popularity metrics, which we used as a proxy for audience reach and influence. Although Apple has not publicly disclosed the full algorithm, available industry reporting indicates that the Mental Health category chart is weighted toward recent download velocity, subscription growth, and listener ratings, with a modest emphasis on completion rate [28]. Focusing on highly ranked podcasts ensured that our sample represented programs with substantial listener engagement and potential societal impact. Our sampling strategy was guided by two primary considerations. First, by prioritizing popularity, we sought to capture podcasts that are more likely to reach broad audiences and shape public discourse surrounding mental health. Second, accessibility was an important factor, as listeners are often guided by visibility and ranking when selecting podcasts, making top-ranked shows a meaningful indicator of audience exposure. From each of the 100 selected channels, three episodes were randomly chosen to serve as the analytical sample. For each channel, the sampling frame consisted of all episodes available in the Apple Podcasts archive at the time of data collection (February–June 2024). Episodes are sorted newest-first, and a randomly generated number determines which one is selected by counting from the top of the list. The selection of three episodes per channel follows precedent in podcast research [29], where three episodes have been used to characterize each show’s structural and stylistic features while keeping the corpus manageable. Across the full sample, this yielded 300 episodes for analysis. This approach provided a balanced representation of each podcast’s content while maintaining a manageable data volume for systematic coding. The titles of all selected channels and episodes, including their Apple Podcasts URLs, are presented in Appendix A.

### Development of the coding scheme

We used a theory-based coding scheme that met the conceptual requirements detailed in the PodCred framework. For the creation of each code in the codebook, we drew the initial categories and definitions from the PodCred framework and organized them into four major categories. The first, *Podcast Content*, captured data related to the substantive aspects of the podcast, including topic, number of hosts and guests, language complexity, and topic delivery. The second, *Podcaster*, included features related to the speaker’s identity, communication style, and demographic attributes. The third category*, Podcast Context*, included elements such as listener feedback, advertisements, endorsements, sponsorships, and the presence of an online store. The fourth category, *Technical Execution*, focused on production quality and audio features, such as background noise, music or sound effects, and recording quality.

Three coders Regina Alcántara (RA), Malee Bedolla (MB), and Kexin (Maggie) Gao are all upper-division undergraduate students and members of the research team, were majoring in communication and health studies and had completed prior coursework in research methods. RA and KG had previously served as research assistants on content analysis projects, providing direct familiarity with systematic coding procedures. All three completed structured training covering the PodCred Framework and the conceptual and operational definitions of each variable in the codebook, training was supervised by the first author Dr. Romy RW, who holds expertise in mental health communication and content analysis. Beyond their academic and methodological preparation, all three coders brought direct experience with the medium under study, each had hosted their own podcast, and RA and Maggie Gao’s channels focused specifically on mental health topics. This combination of methodological training and lived experience with podcasting positioned the coders to engage interpretively with both the structural and thematic dimensions of the content analyzed in this study.

The coders first conducted a pilot coding of five podcast channels, with each coder listening to three episodes per channel before coding the channel as a whole. During the pilot phase, coders focused on refining the theory-driven coding scheme, clarifying code definitions, and ensuring a shared understanding of how the PodCred framework should guide their judgments. The primary goal of this pilot round (*N*_Episodes_ = 15) was to test the scope and applicability of the coding scheme and procedures. Particular attention was given to issues of validity and to identifying the need for additional categories that could better capture the variance in the data. During the development of the codebook, coders derived initial definitions for each code based on the Podcred framework. At the outset, coders carefully reviewed the framework and collaboratively refined the operational definitions of all codes to ensure shared understanding and conceptual clarity. This process involved systematically examining the codebook and verifying that every coder comprehended the meaning and boundaries of each category. Following the explanation of the coding procedures, a pilot coding was conducted in which all coders analyzed the same podcast channels, each containing at least three episodes, over the course of one week. After completing this coding, the coders and I convened to discuss any discrepancies that emerged in the coding process. These discussions focused on clarifying the interpretation of specific codes and locating relevant information within the transcripts.

Several revisions were made following the pilot coding. One of the primary changes involved refining the wording of the codes and their operational definitions. In the original codebook, the response options (or ‘tribes’) consisted of three categories: yes, no, and numerical values. In the revised version, this was expanded to five categories: yes, no, unsure, not applicable, and numerical values. This modification enhanced the accuracy of the data collection process by allowing greater flexibility and preventing coders from forcing observations into overly limited categories. Several new codes were added including podcaster age, sex, sexual orientation, race/ethnicity, and mental health topics. Mental health topics included subcategories such as anxiety disorder, attention-deficit/hyperactivity disorder, autism spectrum disorder, bipolar disorder, borderline personality disorder, depression, eating disorder, obsessive-compulsive disorder, trauma-related conditions, schizophrenia, suicide prevention, and others (specify). Demographic codes were also expanded to include age, gender, sexual orientation, and race/ethnicity. Furthermore, a ‘continuous’ variable was added after each categorical demographic code to capture numerical or range-based data. For example, for the age code, the coder would indicate ‘yes’ or ‘no’ depending on whether all participants in a podcast belonged to the same age group. The subsequent age (continuous) code included defined numerical ranges, and coders would then record the corresponding range number.

### Content analysis

A directed qualitative content analysis [30] was performed. This approach was appropriate because the coding process was guided by an existing theoretical framework (PodCred), which provided initial categories for analysis while allowing for the identification of new themes emerging from the data. For each episode (the unit of analysis), trained coders applied the predefined categories derived from the PodCred framework to systematically analyze podcast content. When data did not fit the existing codes, new subcategories were created inductively to capture novel patterns or nuances specific to mental health podcasting. Through this process, the analysis both validated and extended the PodCred framework, offering a refined understanding of how credibility, authenticity, and communication practices are constructed across mental health podcast contexts.

The final coding scheme consisted of four overarching dimensions, each comprising multiple categories: (1) Podcast Content: Podcast has a strong topical focus, mental health topics, appearance of (multiple) on-topic guests, participation of multiple hosts, same host, use of field report, contains encyclopedic/factual/testimonial information, contains discussion/opinions, contains recommendations/suggestions, podcaster cites sources, podcast maintains its topical focus across episodes, consistency of episode structure, presence/reliability of inter-episode references, episodes are published regularly, episodes maintain a reasonable minimum length, how long the episodes range, fluency/lack of hesitations, speech rate, articulation/diction, use of conversational style, use of complex sentence structure. (2) Podcaster: Podcaster shares personal details, use of broad/creative vocabulary, use of simile, presence of affect, use of invective, use of humor, episodes are succinct, podcaster eponymous, podcaster credentials, podcaster affiliation, podcaster widely known outside the podosphere, age, gender, sexual orientation, race/ethnicity. (3) Podcast Context: Podcaster addresses listeners directly, podcast episodes receive many comments, podcaster responds to comments and requests, podcast page or metadata contains links to related material, podcast has a forum, podcast is a republished radio broadcast, makes reference to current events, podcast has a store, presence of advertisements, podcast has a sponsor, podcast displays prizes or endorsements. (4) Technical Execution: Signature intro/opening jingle, background music, atmospheric sound, editing effects, studio-quality recording/no unintended background noise, feed-level metadata present/complete/accurate, episode-level metadata present/complete/accurate, audio available in high quality/multiple qualities, feed has a logo (logo links to homepage), episodes presented with images, simple domain name, distributed *via* distribution platform, podcast has portal or homepage, reliable downloading.

In line with the procedures of directed qualitative content analysis, exemplars were used to demonstrate how the predefined codes derived from the PodCred framework appeared in the data. Each coder was instructed to upload the episode transcripts and highlight key parts that best represented specific codes. These exemplars served to confirm the accurate application of theory-driven categories, enhance consistency across coders, and support the interpretive validity of the analysis.

### Intercoder reliability

Intercoder reliability was not calculated during the pilot coding, as its primary purpose was to develop and refine the codebook. Following this stage, three coders independently double-coded ten podcast channels, each containing three episodes (*N*_Episodes_ = 30). Percent agreement across all coding categories ranged from 73.3% to 100% (See Table 1), indicating acceptable to high levels of consistency [31]. Percent agreement is reported as the primary reliability index because most coding variables exhibit highly skewed prevalence distributions. Under such conditions, Cohen’s kappa is susceptible to the ‘kappa paradox’, in which chance-corrected coefficients are artificially deflated despite high observed agreement [32, 33], and similar limitations have been noted for other chance-corrected indices. Consistent with Neuendorf’s [34] guidance, percent agreement is reported given the extreme prevalence distributions and the descriptive rather than inferential analytic strategy of the present study. Several codes were excluded due to low intercoder reliability, including background music, episodes maintain a reasonable minimum length, podcast displays prizes or endorsements, podcast episodes receive many comments output, podcast has a sponsor, podcast has a store, podcast is a republished radio broadcast, race, sexual orientation, use of field report, podcaster responds to comments and requests, and ID3 tags used. The remaining coding was randomly divided among the three coders, including 85 podcast channels (*N*_Episodes_ = 255).

**Table 1:** **%** agreement across coding categories

Category	Percentage agreement	Percentage presence (*N* = 100)
***Podcast content***		
Podcast has a strong topical focus	87	100
Appearance of on-topic guests	87	43
Participation of multiple hosts	100	21
Same host	100	95
Contains encyclopedic information	87	81
Contains discussion	87	91
Contains recommendations	100	95
Podcaster cites sources	73	50
Podcast maintains its topical focus across episodes	87	100
Consistency of episode structure	100	97
Presence/reliability of inter-episode references	87	34
Fluency/lack of hesitations	87	97
Speech rate	100	100
Articulation/diction	100	100
Use of conversational style	87	84
Use of complex sentence structure	87	40
***Podcaster***		
Podcaster shares personal details	87	85
Use of broad/creative vocabulary	100	97
Use of simile	100	74
Presence of affect	100	83
Use of invective	87	18
Use of humor	73	48
Episodes are succinct	73	98
Podcaster eponymous	100	90
Podcaster credentials	73	93
Podcaster affiliation	100	91
Podcaster widely known outside the podosphere	73	78
Age	73	45
Gender	73	45
***Podcast context***		
Podcaster addresses listeners directly	73	92
Podcast page or metadata contains links to related material	73	93
Podcast has a forum	73	71
Makes reference to current events	100	88
Presence of advertisements	87	87
***Technical execution***		
Signature intro/opening jingle	73	86
Atmospheric sound	73	25
Editing effects	87	87
Studio-quality recording/no unintended background noise	87	98
Feed-level metadata present/complete/accurate	100	100
Episode-level metadata present/complete/accurate	100	98
Audio available in high quality/multiple qualities	87	14
Feed has a logo	87	95
Episodes presented with images	87	32
Simple domain name	100	98
Distributed *via* distribution platform	100	99
Podcast has portal or homepage	100	99
Reliable downloading	100	99

## Results

The results of the content analysis are summarized in Appendix B. In general, the results of the directed content analysis reveal clear patterns in the characteristics of top-ranked mental health podcasts, providing insight into the specific features that appear to attract and sustain listener engagement. Across the four major dimensions of the PodCred Framework: Content**,** Podcaster**,** Podcast Context**,** and Technical Execution. In what follows, we will discuss each category according to its prevalence in the data and enrich the analysis with quotes.

### Content

#### How long the episodes range

Across the 100 podcast episodes coded, episode lengths varied substantially, ranging from 1 minute to 173 minutes (*Mean* = 44.6 minutes, *Median* = 43 minutes). The analysis of episode lengths also shows that podcast creators tend to favor medium-length episodes, with most content falling close to the 40–45 minutes range. Short-form episodes under 20 minutes were less common but appeared across several shows.

#### Episodes are published regularly

Across the podcast collection, publication schedules demonstrated substantial variability, though weekly releases were by far the most common pattern. A large majority of shows adhered to a regular weekly schedule, with additional programs publishing semiweekly or biweekly. A smaller number followed more intensive release patterns, including daily, every-two-days, or every-three-days schedules. In contrast, several podcasts exhibited irregular or sporadic release intervals, and a few provided insufficient information to determine a consistent schedule. Overall, despite the presence of less frequent or inconsistent posting patterns, the dataset was characterized primarily by podcasts that publish on a predictable and recurring weekly basis.

#### Topical focus and structural consistency

Nearly all podcasts in the sample (100%) maintained a strong topical focus and stayed consistent in structure (97%), suggesting that listeners value predictability and coherence in mental health content. Episodes also regularly preserved continuity across episodes, with 100% demonstrating reliable inter-episode references. This continuity may cultivate a sense of narrative progression or cumulative learning, which is particularly important in health-related educational content.

#### Delivery style

It was also noted that most popular podcasts among listeners used a conversational style (84%). This format facilitated a parasocial relationship between listeners and the podcaster, supporting literature around the connection which develops through the intimacy of podcast conversational style [16]. However, this dataset suggests that such formats might not be necessary for popularity; instead, consistent solo-hosted educational content may be equally or more compelling to audiences in this domain.

#### Types of content

The content most included recommendations or suggestions (95%), discussion or opinion sharing (91%), and encyclopedic or factual information (81%) (see Table 2 Illustrative Quotes).

**Table 2:** Illustrative quotes

Dimension / Feature	Illustrative quote	Podcast episode
Content: Recommendations/Suggestions	You just worry about all of those things. But first, you have to take care of you. You have to take care of you first. And so I have been able to do that with the help of friends like you and not surrender my story to pain.	In the Light with Dr. Anita Phillips (Episode: *Power Moves w Sarah Jakes Roberts.*)
	Sharing our stories with others and imposing meanings on them is exactly what creates communities of healing and hope.	Back From the Borderline (Episode: *childhood emotional neglect part 11: Healing*)
Discussion/Opinion Sharing	I think so often when people are in leadership positions or as the head of their home or the one that’s supposed to be strong in whatever circumstances . . . you feel like you can’t let down all the people that are under you.	The Heare Brotherhood (Episode: Nathan Tanner: From panic attack to triathlons, principles from his book The Unconquerable Leader, Mastering the Internal and External Game)
	That’s the great thing about family—no matter who you change into, what you grow into, whatever you become, they know every version of you before. And there’s an essence, right, that is the same.	That Conversation with Tarek Ali (Episode: Whole lotta SEX—Healing Body Trauma, Sexual Agency, Growing Beyond Judgement)
	But I think that there is a person that goes into remission, and they haven’t had disease ‘activity’ for six years, and they want to have a child—then they say, go for it.	Real Pod (Episode: I Had a Baby *via* Surrogate! Mallory Goldman’s Motherhood Journey, Autoimmune Disease, & Supportive Hubby)
Content: Encyclopedic/Factual	Biologically, anger’s role, one, is to highlight a problem. So with anger, there’s a release of norepinephrine: it’s very alerting, it’s energizing.	Being Well with Forrest Hanson and Dr. Rick Hanson (Episode: *Anger, Repression, and Self-Expression*)
	Pew Research specifically talked about from 2018 to 2021, we saw suicide rates among Black youth grow at a faster rate than any other racial group.	Therapy for Black Girls (Episode: *Tending to Youth Mental Health*)
	The gene codes for the serotonin transporter, which, as the name implies, helps transport serotonin back into the neuron in the brain. The gene comes in two major alleles, or forms: the short arm (S/S) allele and the long arm (L/L) allele.	Carlat Psychiatry (Episode: *Throwback: The Bright Side of the Serotonin Gene*.)
Podcaster: Self-disclosure	I was so desperate for connection that I did dumb things trying to get pseudo-connected. I ate too much, I drank, I used pornography, and alcohol made me anxious.	The Dr. John Delony Show (Episode: *I Was on OnlyFans and I Regret It*)
	I used to lie all the time. About everything. I used to steal all the time. I was just a punk kid.	
	I fought this disease for 14 years before remission. I’ve been dealing with it since childhood.	Trauma Is Expensive (Episode: *Breaking Free from Emotional Chains: The Quest for Self-Love and Mutual Respect*)
	Cancer was my whole life. I fought it for so long, and on top of all the other trauma, I didn’t know who I was.	
	Just like dating, friendships can fall apart. Trust me: I’ve been that person, I’ve done that.	The Psychology of Your 20’s (Episode: *Disliking Your Friend’s Partner*)
Podcaster: Creative vocabulary	They built something called a ‘ludic loop.’ It comes out of video gaming and refers to a story arc that has no development.	Big Dating Energy (Episode: *Burn the Haystack with Dr. Jennie Young*)
	You’re out there grinding your goals, being productive, and that’s okay.	The Mindset Babe (Episode: *And It’s OK!!*)
Podcaster: Humor	As long as we keep her away from the knives, this is going to be good TV.	Sober Mom Podcast (Episode: *SobrieTEA: The Vanderpump Rules Reunion & the End of Carl and Lindsay.*)
	Now we’ll get to meet drinking Janet, because this was just pregnancy-hormones Janet.	
	I like living on the pink cloud. The pink cloud is great: it’s the thunderbolts that come along and smack you down.	Recovery Elevator (Episode: Advice for the Newly Sober.)
Podcaster: Profanity or blunt language	I walked past the nervous-looking stewardess, smelling no doubt of deodorant and my own shit: there’s no end to the things we do for drugs.	Dopey (Episode: Hunting Serial Killers Operating Under the Cloak of America’s Opioid Epidemic, Billy Jensen, Alcohol, Recovery, Chris Paulson.)
	We’re sick and tired of being sick and tired, tired of the shit. It’s exhausting holding addiction together and functioning.	Recovery Elevator (Episode: NA Beers)
	You went to a strip club, remember this dipshit did?	The Heare Brotherhood (Episode: Jimmy Rex: Starting We are The They, Creating True Friendship, Living in Integrity and Holding Men Accountable, Growth in Adversity.)
Context: Second-person language	In today’s episode, I’m talking to you about seven easy practices you can use to quiet your anxiety.	The Anxiety Coaches (Episode: 7 Easy Practices to Quiet Your Anxiety)
Context: Direct address	Every Monday, I introduce you to a guest whose story and expertise can inspire you to think, feel, and do your best.	Mentally Stronger (Episode: Overcoming the Fear of Feeling with Author Jennie Allen)
Context: Audience participation	If you want to be on the show, give me a buzz at 1–844–693-3 291 or go to johndelony.com/ask.	The Dr. John Delony Show (Episode: My Husband Spent $80 000 on Porn)
	You can send me a voicemail by visiting backfromtheborderline.com.	Back From the Borderline (Episode: Childhood Emotional Neglect Part 11: Healing)
	We are taking your questions live during our Sunday episode tapings in these lightning rounds.	The Badass Counseling Show (Episode: Q&A: Narc Boyfriend, Dysfunctional Relationship, and More)
Context: Current events	During the pandemic, our community experienced vicarious trauma around racial issues: one thing after another, and we can’t underestimate its impact on young people.	Therapy for Black Girls (Episode: Tending to Youth Mental Health)
	Spurred by the ‘Me Too’ movement, there’s been a crackdown on the misuse of confidentiality clauses, including the Speak Out Act of 2022 limiting NDAs in sexual harassment disputes.	Back From the Borderline (Episode: Exposing Miss USA’s Dark Secrets: Title Holder Resignations, Misogyny, Corruption, and Everywhere Like Such As)
	The show notes link to resources, international and local, so social well-being becomes part of positive change, not just saying ‘Black Lives Matter’ or ‘Stop AAPI Hate,’ but learning more and taking action.	Sleep With Me (Episode: Wildee Wonga Tour)

#### Guest and host composition

Most episodes featured a single, recurring host (95%). In contrast, only 21% included multiple hosts, and just 43% included on-topic guests. This pattern diverges from earlier findings in mental health podcasting that conversational-style interviews are most prevalent among listeners [10]. The interpretive implications of this divergence will be discussed in the Integrative Discussion.

#### Language style

Podcasters overwhelmingly displayed strong articulation (100%) and spoke fluently with minimal hesitation (100%). Speech rates were generally clear (100%). This indicates that accessibility of language and ease of listening which are the key components of PodCred’s content and style criteria. Only 40% used complex sentence structures, suggesting that most podcasters aimed to keep content digestible rather than intellectually dense, consistent with the educational goals of mental health literacy promotion.

### Podcaster

#### Self-disclosure and emotional expression

The majority of podcasters shared personal details (87%) and used affective cues such as emotional tone (83%). Podcasters frequently disclosed deeply personal experiences, including past behaviors, regrets, health struggles, and identity challenges. This reflects the importance of relational warmth and authenticity, qualities that prior scholarship has connected to parasocial bonding, listener trust [16], and stigma reduction [35]. High self-disclosure may foster trust and parasocial closeness, both of which have been linked to listeners’ increased sense of community [14] and reduced stigmatizing attitudes toward mental health [10]. (see Table 2 Illustrative Quotes).

#### Credibility markers

Ninety-three percent of podcasters displayed explicit professional credentials, and 91% indicated an affiliation, such as a clinical practice, academic institution, or professional organization. This finding supports the PodCred Framework’s emphasis on expertise and trustworthiness as defining factors of podcast attractiveness. In contrast, only 45% of podcasters were widely known outside the podosphere, suggesting that celebrity is not a prerequisite for popularity within the mental health genre. Rather, professional credibility appears to hold more relevance than public fame. For example, the host of *Feeling Good Podcast*, Dr. David Burns, is an Emeritus Clinical Professor of Psychiatry at Stanford University School of Medicine. Dr. Russell Ramsay, a guest on the podcast of *ADDitude ADHD Experts*, is a clinical psychologist and former professor at the University of Pennsylvania’s Perelman School of Medicine. The host of *Mayim Bialik’s Breakdown* holds a PhD in neuroscience, and her popularity is plausibly amplified by her well-established acting career, including her long-running role as neuroscientist Amy Farrah Fowler on the popular sitcom *The Big Bang Theory*, so her case represents a hybrid of professional credibility and pre-existing public fame.

#### Communication style

Most podcasters used creative vocabulary (97%), humor (48%), and occasional invective (18%). Humor and similes were variably used, suggesting that stylistic creativity is helpful but not uniformly necessary across top-ranked shows. These stylistic choices reflect the informal, intimate tone characteristic of podcast storytelling. Humor, even when used sparingly, may help diffuse tension when discussing heavy topics like trauma or grief.

Podcasters frequently used vivid or unconventional language to enhance narrative engagement and conversational flow. (see Table 2 Illustrative Quotes)Humor was often used to soften difficult subject matter, create relatability, or reframe stressful situations. (see Table 2 Illustrative Quotes).

A smaller subset of podcasts used profanity or blunt language, often to convey authenticity or emotional intensity. (see Table 2 Illustrative Quotes)**Podcast Context.**

#### Audience engagement and interactivity

A large majority of podcasters addressed listeners directly (92%), a strategy known to strengthen perceived intimacy and interpersonal connection [36]. Several approaches have been used to encourage audience engagement and participation.

Podcasters frequently spoke to listeners using second-person language, invitations, and inclusive framing (see Table 2 Illustrative Quotes).

Other podcasts used direct address to frame ongoing programming and expectations. For instance, Mentally Stronger highlighted regular audience-oriented content (see Table 2 Illustrative Quotes).

Beyond conversational address, some podcasts invited active audience participation through calls, voicemails, or live questions (see Table 2 Illustrative Quotes).

Live interactivity also appeared in some formats, as illustrated by The Badass Counseling Show (see Table 2 Illustrative Quotes).

#### Balancing social context

**References to current events appeared often (88%), indicating that mental health podcasts ground their content within broader social contexts, such as national crises and social movements. This reflects flexibility in the level of interactivity or real-time engagement expected from mental health podcast audiences (see Table 2 Illustrative Quotes)**.

#### Commercial elements

Most podcasts included links to related materials (93%) and incorporated advertisements (87%). The high rate of advertising supports the notion that top mental health podcasts often operate as monetized media products, which may introduce tensions between authenticity and commercialization.

### Technical execution

#### High baseline production quality

Technical quality was consistently high across the sample, indicating that production standards have become a baseline expectation among mental health podcasts. Nearly all podcasts featured studio-quality audio (98%), accurate metadata (100% at the feed level; 98% at the episode level), reliable downloading (99%), and were hosted on a distribution platform with an associated homepage (99%). These findings suggest that listeners expect seamless access and professional sound quality as standard features.

#### Optional aesthetic enhancements

While foundational production elements were nearly universal, optional aesthetic features varied. Signature intros were present in 86% of podcasts, editing effects in 87%, and atmospheric sounds in 25%. This pattern suggests that although polish is valued, additional embellishments are not essential for listener engagement. Episodes presented with images were relatively rare (32%), and only 14% of feeds featured feed-level logos, indicating that visual branding at the episode level is largely supplementary.

#### Branding and presentation

Most feeds included logos (95%), signature intros (86%), and consistently used distribution platforms (99%). Only 32% included episode images indicating that while branding is important, visual episode-level customization is not.

### New codes

#### Mental health topics

The qualitative content analysis showed that podcasts most frequently focused on common mental-health concerns such as anxiety, depression, stress, and trauma. Many episodes also explored a broad range of related issues including insomnia, addiction, alcoholism, grief, toxic relationships, emotional regulation, and self-development. Although less common, some episodes addressed topics like ADHD, autism, and bipolar-related experiences, typically woven into broader conversations about identity and coping. Overall, the dataset highlights that mental-health podcasts tend to prioritize widely relatable emotional challenges while incorporating a diverse mix of personal well-being themes.

#### Age

Based on our coding, the podcasters represented a range of age groups, with young adults (18–39 years) and middle-aged adults (40–59 years) being the most common. Older adults (60+ years) appeared less frequently, and children/adolescents (under 18) were rarely reported. Several podcasters indicated multiple applicable age ranges or were unsure of their classification. Overall, this distribution suggests that mental health podcasts are primarily created by adults within the young and middle-aged adult categories, reflecting a workforce that is both experienced and digitally engaged.

#### Gender

The podcasters represented a mix of genders, with females and males being the most common. Many podcasts were co-hosted by both female and male podcasters, reflecting a collaborative dynamic, while solo podcasters tended to be slightly more often female (36, 45.6%), compared to 15.2% male-led (12) and mixed gender (30, 38.0%) (see Appendix B). There were no consistent indications of non-binary or other gender identities reported in the sample. Overall, these findings suggest that mental health podcasts are predominantly produced by adults identifying as female and male, with gender-diverse representation either limited or unreported. We discuss the implications of this distribution in the Integrative Discussion below.

### Comparative analysis

Because every podcast in the sample is, by construction, ‘popular’ (ranked in Apple’s Top 100), variation within the ranking of podcasts provides a useful descriptive comparison. We compared the top 10 podcasts with the bottom 10 podcast on mental health topic focus, host credentials, delivery style, and production quality (see Table 3).

**Table 3:** Comparative analysis

Category		Top 10 (%)	Bottom 10 (%)	Difference
**Topic Focus**	Strong topical focus	100%	100%	0
	Cites sources	100%	20%	80
	Maintains topical focus across episodes	100%	100%	0
	Episode structure consistency	100%	90%	10
**Host Credentials**	Documented host credentials	100%	100%	0
	Host widely known outside podosphere	100%	30%	70
**Delivery Style**	Fluency/lack of hesitations	100%	100%	0
	Articulation / diction	100%	100%	0
	Conversational style	100%	90%	10
	Complex sentence structure	50%	10%	40
**Production Quality**	Atmospheric sound	90%	20%	70
	Editing effects	100%	80%	20
	Signature intro / opening jingle	70%	100%	30
	Studio quality recording	100%	100%	0
	Feed-level metadata complete	100%	100%	0
	Episode-level metadata complete	100%	100%	0

***Mental Health Topic Focus.*** Both groups demonstrated uniformly strong topical focus and consistent maintenance of that focus across episodes, indicating that topical coherence alone does not differentiate higher from lower ranked podcasts. However, a substantial difference emerged in source citation behavior: all top 10 podcasts cited sources compared to only 20% of bottom 10 podcasts. This finding suggests that epistemic credibility and evidence-grounding, rather than topical focus *per se*, may be more closely associated with podcast popularity in the mental health space.

***Host Credentials.*** Documented host credentials were uniformly present in both groups, meaning formal credentials alone do not account for the popularity gap between ranked groups. A more meaningful distinction emerged in broader public recognition: all top 10 podcast hosts were coded as widely known outside the podosphere compared to only 30% of bottom 10 hosts. This pattern suggests that pre-existing audience reach and public visibility may amplify discoverability and listenership in ways that formal credentials alone do not.

***Delivery Style.*** Fluency and articulation were uniformly high across both groups, indicating that baseline speech quality is a necessary but not sufficient condition for popularity. The most notable delivery difference was in complex sentence structure, present in 50% of top 10 podcasts compared to only 10% of bottom 10 podcasts. This may reflect a more substantive and intellectually engaging communication style among higher ranked podcasts, consistent with audience preferences for substantive intellectual engagement in mental health content.

***Production Quality.*** Studio recording quality and metadata completeness were comparable across both groups. The most striking production difference was in atmospheric sound, present in 90% of top 10 podcasts but only 20% of bottom 10 podcasts. This suggests that immersive audio design, including ambient soundscapes and intentional sonic environments, may be an underappreciated marker of production investment associated with higher ranked mental health podcasts. Notably, bottom 10 podcasts were more likely to feature a signature intro or opening jingle (100% versus 70%), suggesting that surface-level production cues do not substitute for the deeper sonic craft associated with atmospheric design.

In conclusion, these descriptive comparisons reveal that the characteristics most meaningfully associated with podcast ranking are not those related to topical focus or basic production standards, which are uniformly present across both groups, but rather those reflecting credibility signals (source citation, public recognition), linguistic sophistication (complex sentence structure), and immersive audio design (atmospheric sound).

### Integrative discussion

The results suggest that mental health podcasts gain traction through a combination of credibility, consistency, and connection, a triad that maps closely onto the PodCred Framework. Credibility is established through professional credentials, accurate information, and reliable production quality. Consistency emerges through strong topical focus, structural regularity, and predictable communication style. Connection is fostered through personal disclosure, affective delivery, and direct address to listeners. Interestingly, several indicators commonly discussed in prior literature, such as hosting celebrity guests [37] or offering highly interactive forums [38] were not uniformly present across the most popular podcasts. These patterns provide empirical support for the PodCred Framework while also extending it by identifying which surface-level cues are most consequential within the mental health domain.

Read through the lens of parasocial relationship theory, the high prevalence of single-host formats (95%), personal self-disclosure (87%), direct listener address (92%), and emotional affect (83%) is theoretically interpretable rather than merely descriptive. Each of these features corresponds to a documented antecedent of parasocial bonding [15, 16, 18]: a stable, recurring on-air persona, persona-initiated self-disclosure, second person address to the audience, and emotional expressiveness. Across the four domains, within the mental health domain, parasocial intimacy may operate as a near-necessary condition for podcast success. This pattern both confirms and refines prior podcasting studies. It confirms earlier work that identified parasocial relationships as central to podcast listening [16]. It refines that work by suggesting that the parasocial mechanism in mental health podcasting is concentrated in solo-host, personality-driven formats rather than in the interview-with-lived-experience format that prior studies have emphasized [10].

From a U&G perspective, the consistency of structural and stylistic features across the sample suggests that listeners select these podcasts to satisfy a relatively focused gratification profile, including information-seeking, emotional support, and parasocial companionship [21, 22]. The relative scarcity of multiple-host or guest-driven formats may reflect a listener preference for intimate, single-voice delivery when the primary need is emotional regulation rather than information acquisition.

The descriptive distribution of episode length, clustering at 30 to 60 minutes is consistent with the durations listeners report as ideal in previous research [39]. Combined with the dominance of weekly release schedules and 97% structural consistency across episodes, this pattern aligns with what U&G research labels ritualized media use, when listeners can predict the format, timing, and duration of a program, it is more easily integrated into daily routines as habitual, schedule-driven consumption [40, 41]. This regularity, in turn, creates the conditions under which parasocial bonds develop and deepen through repeated, intimate exposure to a consistent host persona [16, 17]. For practitioners and creators, the implication is that predictable cadence and a narrow length corridor may matter more than format experimentation. This nuances the more open-ended PodCred indicator of ‘reasonable minimum length,’ suggesting that within the mental health domain the practical corridor is considerably narrower than the framework itself implies.

The credibility profile we observed both confirms and contradicts earlier work. Confirming the PodCred Framework, professional credentials (93%) and institutional affiliation (91%) are nearly universal among top-ranked mental health podcasters, paralleling podcast research that identifies speaker credentials and source trustworthiness as central to listener preference and content evaluation [24]. Contradicting earlier listener survey research in which audiences reported that discussions featuring professionals and people with lived experience were among the most valued content features of mental health podcasts [10], only 21% of our sample featured multiple hosts and only 43% featured on-topic guests. This divergence has two plausible explanations. First, the mental health niche may have matured into a host-driven genre in which the host’s own credentials and emotional availability matter more than the presence of guests, an interpretation consistent with our observation that 95% of episodes featured a single, recurring host. Second, the platform algorithm itself may favor host-driven shows, as solo-hosted podcasts can release more consistently because they are not dependent on guest scheduling, and data suggest that release consistency is associated with stronger performance [28]. *Mayim Bialik’s Breakdown* illustrates this convergence well. Bialik’s doctoral credentials in neuroscience align with the credibility expectations that characterize the mental health podcast landscape, while her celebrity recognition likely lowers the barrier to initial discovery by drawing on parasocial familiarity established well before a listener engages with the content. When both converge in the same host, the resulting popularity may reflect the combined weight of two distinct selection mechanisms rather than either one alone. Whether such host-credentialed celebrity hybrids represent a meaningful subtype within the genre is a question worth pursuing in future work, particularly through audience reception methods or experimental designs that can separate these two sources of appeal.

One of the most striking findings of the analysis is that 87% of top-ranked mental health podcasts incorporated advertising, and 93% included links to related materials. These rates raise meaningful concerns for health communication ethics that extend beyond what the descriptive PodCred Framework can address. First, the saturation of advertising on platforms dedicated to mental health blurs the boundary between psychoeducational content and commercial speech, a boundary that professional and scholarly communities have identified as ethically significant. When advertising appears within a parasocial relationship that listeners experience as intimate and trustworthy, the persuasive impact of any embedded endorsement is plausibly heightened. Empirical research on podcast advertising specifically found that a parasocial relationship with a podcast host decreased listeners’ evaluative persuasion knowledge about promotional messages, which in turn enhanced their intentions to seek more information about the advertised brand, suggesting that the trust generated through parasocial bonds may lower critical scrutiny of commercial content [42]. This risk is compounded by evidence that health-related influencer content frequently promotes products in ways that are predominantly benefit-focused and underreport potential harms [43]. Second, the prevalence of related-material links suggests a content economy in which the parasocial relationship becomes monetizable through downstream sales of courses, supplements, books, and clinical services. Although advertising and ancillary revenue are important for podcast sustainability, the absence of consistent disclosure norms and any structural separation between editorial and sponsored content creates conditions in which commercial and therapeutic functions become difficult for listeners to distinguish. Explicit disclosure standards and clearer ethical guidelines tailored to mental health podcasters are therefore warranted. Professional organizations such as the American Psychological Association and the American Counseling Association are well positioned to articulate such norms, and doing so would extend PodCred’s context indicators beyond descriptive analysis into a normative framework suited to the specific ethical demands of health communication.

The gender distribution among top-ranked podcasters was uneven, with female podcasters modestly overrepresented and gender-diverse podcasters absent. The absence of non-binary and transgender voices in the Apple Top 100 has clear implications for representation, since gender-diverse audiences face distinct mental health challenges [44] and may benefit from podcasters whose own gender identities reflect their experience. Listener gratifications related to identification and social integration [19] may therefore be less well met for these audiences within the current Top 100, pointing to a structural gap that creators, platforms, and recommendation algorithms could address.

These findings carry theoretical, methodological, and practical significance. Theoretically, the integration of PodCred with parasocial relationship theory and U&G Theory reconceptualizes the function of the framework’s core indicators, rather than serving as neutral descriptive features, the surface-level cues PodCred catalogs operate as antecedents of parasocial bonding and as structural fulfillments of specific listener gratifications. This reconceptualization elevates PodCred from a descriptive inventory to a theoretically grounded account of the mechanisms through which observable podcast characteristics translate into audience appeal. Methodologically, this study represents the first directed qualitative content analysis to apply PodCred within a mental health podcast context and the first to employ Apple’s Top 100 podcast as a popularity criterion in this domain, establishing a replicable methodological template for future genre-specific scholarship. From a practical standpoint, the findings hold implications for multiple stakeholders. Podcast creators would benefit from prioritizing solo-host formats, consistent release cadences, and emotionally accessible delivery styles. Clinicians considering podcast referrals should attend to host credentialing, topical coherence, and the transparency of advertising disclosures. Platform designers, meanwhile, should recognize that the underrepresentation of gender-diverse voices among highly rankedpodcasts reflects structural conditions that algorithmic recommendation systems are positioned to help redress. Finally, where the findings strain against PodCred’s existing architecture, they illuminate a substantive limitation, a domain-general indicator set may systematically underweight parasocial cues in genres where emotional intimacy constitutes the primary gratification listeners seek. Cross-genre comparative studies would offer the most direct empirical test of whether this pattern extends beyond the mental health context.

## Conclusions

This study used a directed qualitative content analysis guided by the PodCred Framework to evaluate the characteristics of top-ranked mental health podcasts. The findings have several implications. Theoretically, the results support the applicability of the PodCred Framework while identifying areas for refinement, including the incorporation of podcaster demographic characteristics and clearer distinctions across mental health topic categories. Methodologically, this study is the first to apply a directed qualitative content analysis to mental health podcasts, offering a foundation for future work examining digital mental health communication. Practically, the findings underscore the need to balance expertise with relatability in mental health communication. Practitioners, educators, and organizations seeking to leverage podcasts should consider formats that foreground authentic storytelling, empathetic dialogue, and conversational language alongside evidence-based information. For creators developing mental health content, maintaining consistency across episodes, providing clear topic focus, and adopting emotionally engaging delivery styles appear crucial for sustaining audience interest. Moreover, high-quality production and coherent branding now function as baseline expectations for podcast credibility. In this context, integrating actionable recommendations, relatable narratives, and accessible language may enhance listener engagement, perceived trustworthiness, and long-term loyalty. Together, these insights highlight the evolving landscape of mental health podcasting and point toward best practices for creating digital mental health communication.

### Limitations

Several limitations should be acknowledged. First, the sample was restricted to the Apple Podcasts Top 100 mental health rankings. This focus limits the generalizability of the findings, as other major platforms (*e.g.* Spotify, YouTube) may highlight different podcasts, use different ranking algorithms, or reflect different audience demographics. Access was further constrained by the requirement of an Apple device, which may exclude perspectives from listeners who do not use iOS or rely on alternative podcast ecosystems. Second, the analysis included only English-language podcasts. This language restriction introduces bias and limits applicability to global podcasting landscapes where mental health communication norms may differ. Third, the reliance on publicly available podcast materials (audio, descriptions, websites, and metadata) restricts the analysis to content intentionally published by creators. This excludes behind-the-scenes production decisions, audience analytics, and intentions that may shape content but are not visible to external coders. Fourth, although directed qualitative content analysis provides systematic structure, it remains subject to the interpretive nature of coding. Finally, focusing exclusively on top-ranked podcasts may bias the sample toward productions with higher visibility, stronger branding, or greater institutional support, potentially underrepresenting independent or emerging podcasts that may adopt different communication strategies.

## Supplementary Material

Supplementary_materialcyag027

## Data Availability

The data underlying this article are available in the article and in its online supplementary material.
