# Correction: A content analysis of the characteristics of the popular mental health podcasts

**DOI:** 10.1093/her/cyag033

**Published:** 2026-10-14

This is a correction to: Romy RW, Regina A A Alcántara, Malee Bedolla, A content analysis of the characteristics of the popular mental health podcasts, Health Education Research, Volume 41, Issue 5, October 2026, cyag027, https://doi.org/10.1093/her/cyag027

The following change has been made to the originally published paper. The names of the first author were erroneously tranposed and are now corrected to read: “Romy RW” isntead of: “R W Romy”.

