# Supplementary material for: A content analysis of the characteristics of the popular mental health podcasts
Source: Health Educ Res. 2026 Sep 10;41(5):cyag027. doi: 10.1093/her/cyag027 (PMC13561107; doi:10.1093/her/cyag027)
Supplement: Supplementary file 1 [file supplementary_materialcyag027.zip › Appendix A Mental Health Podcast Episodes.doc]

**Supplemental Table 1**

*Top 100 Mental Health Podcasts and Sampled Episodes*

| **Podcast Channel** | **Episode#1** | **Episode#2** | **Episode#3** | **Apple Podcasts URL** |
| --- | --- | --- | --- | --- |
| On Purpose | Ed Mylett:#1 Habit to Become an Expert at Time and Energy Management | 3 ways to build inner confidence & learn to trust yourself instead of comparing yourself to others | 2 way your ‘icks’ are ruining your dating life & #1 way to date better with Jared Freid and Jordana Abraham | <https://podcasts.apple.com/us/podcast/on-purpose-with-jay-shetty/id1450994021> |
| Ten Percent Happier | How to stay calm no matter what’s happening \| Sebene Selassie and Jeff Warren | Science-Based Tools for When You’re Stressed, obsessed, or overthinking \| Dr. Jenny Taitz | How not to ruin your relationships \| Drs. John & Julie Gottman | <https://podcasts.apple.com/us/podcast/10-happier-with-dan-harris/id1087147821> |
| Pursuit of Wellness | Girl Chat: Acne Safe Makeup, Why I drink aloe juice, supplements for fertility & giving y’all advice 2/fi. | How to Live a life without to limits and transform your reality with tom McCarthy | Why protein & Muscle are so important + Ozempic (he truth) weight lifting & fat loss w/ dr. Gabrielle Lyon | <https://podcasts.apple.com/us/podcast/pursuit-of-wellness/id1655979078> |
| These Fukken Feelings Podcast | The Odyssey of Inner Peace Amanda Kirkland’s Chronicles of Transformation | The Gentle Art of Not Giving a F*ck Button Pushers Unpacked-Heather Warren | Finding light in Darkness: Betsy Ross’s Journey & Mission | <https://podcasts.apple.com/us/podcast/these-fukken-feelings-podcast/id1580667655> |
| The Doctor John Delony Show | Sex conversation with my wife are uncomfortable | I was on OnlyFans and I regret it | My husband spent $80,000 on Porn | <https://podcasts.apple.com/us/podcast/the-dr-john-delony-show/id1527609854> |
| Chasing Life | Lindy west on what we’re getting wrong about weight | Is obesity really a disease? | The Truth about menopause and weight gain | <https://podcasts.apple.com/us/podcast/chasing-life/id1501029683> |
| Mayim Bialik’s Breakdown | Stuck emotions are making you sick, with Dr. Peter A. Levine! | I was Enmeshed with my mom, with Brett Gelman | Why Bad boy’s feel good, with Dr. Stephen Porges! | https://podcasts.apple.com/us/podcast/mayim-bialiks-breakdown/id1546456269 |
| The Psychology of your 20s | How to get your spark back | Disliking your friends partner | Social media is ruining my mental health | <https://podcasts.apple.com/us/podcast/the-psychology-of-your-20s/id1573710078> |
| UnF*ck Your Brain | Self-help when the world is burning (Feministe Mindset Principles series Ep2) | Greatest Hits: Ghost Patterns-anxiety, fixation & survival | Why sometimes success doesn’t feel good: a conversation with Laura Gassner Otting | https://podcasts.apple.com/us/podcast/unf-ck-your-brain-feminist-self-help-for-everyone/id1229434818 |
| Sleep With Me | Well Met\| Alba Salix S1E1\|Listener fav #868 | Tiki Transistor Hut\|Multiplex Ep5 | Wildee Wonga Tour | <https://podcasts.apple.com/us/podcast/sleep-with-me/id740675898> |
| Big Dating Energy | Selfish Forgiveness with Gabrielle Stone | Busy Philipps Has a Lot to Offer | Burn the Haystack with Dr. Jennie young | <https://podcasts.apple.com/us/podcast/big-dating-energy/id1726065949> |
| Trauma is Expensive | Transforming pain into progress in the shadow of trauma | Breaking free from emotional chains the quest for self-love and mutual respect | The unspoken storm: a child’s silent struggle beside a mother’s battle with cancer | <https://podcasts.apple.com/us/podcast/trauma-is-expensive/id1556750156> |
| Calm it Down | Praying for health over a candy bar: making real change happen | Making peace with your body: ending the battle against yourself | The firefly within: healing childhood wounds | <https://podcasts.apple.com/us/podcast/calm-it-down/id1560836226> |
| Why Not Me The World | John of showboat tv: orchestrating inclusion and celebrating diversity through music and autism engagement | Dr. Ashley Ruba’s Candid Reflections on life with Autism and ADHD | Katie Griswold: Chasing Dreams and championing Autism Advocacy on the pageant stage | <https://podcasts.apple.com/us/podcast/why-not-me-the-world/id1200361736> |
| Therapy for Black Girls | Session 355: The work of Death Doulas | Session 354: ICYMI, Pregnancy,& postpartum concerns | Session 353: Art Therapy 101 | <https://podcasts.apple.com/us/podcast/therapy-for-black-girls/id1223803641> |
| Therapy and Theology | S6E6 If you’re afraid to go to counseling | S6E5 Can we control what we think about | S6E4 Why do I feel so angry | <https://podcasts.apple.com/us/podcast/therapy-and-theology/id1641587501> |
| The One You Feed | Understanding the Truth about obesity and weight loss drugs | How to unlock your inner greatness with George Mumford | Understanding how the brain works with Lisa Feldman Barrett | <https://podcasts.apple.com/us/podcast/the-one-you-feed/id792555885> |
| Transform | FORM’s New trainer, benefits of gym workouts and starting your wellness journey with Brynley Joyner, | The power of the words we speak to ourselves | Nutrition tips + navigating supplements with JSHealth’s Jessica Sepel | [https://podcasts.apple.com/us/podcast/transform/id1531882104](https://podcasts.apple.com/us/podcast/transform/id1722184073) |
| Wellness Her Way | Tips to support liver detoxification, healing from Borderline Personality disorder… | Living intuitively for your body, healing your relationship with fitness | Identifying PCOS & endometriosis, advocating for your health | <https://podcasts.apple.com/us/podcast/wellness-her-way/id1551992770> |
| Affirmations For Black Girls | Happiness is a choice. CHOOSE HAPPY | Finding purpose in an unprecedented pause | Embracing flexibility & focus in this new season | <https://podcasts.apple.com/us/podcast/affirmations-for-black-girls/id1579248838> |
| The Minimalists Podcast | the presence of absence | rental culture | simplify simplify simplify | <https://podcasts.apple.com/us/podcast/the-minimalists/id1069757084> |
| Calm For Sleep | journey into sleep | arctic cruise | the beauty of winding down | <https://podcasts.apple.com/us/podcast/calm-for-sleep/id1503023709> |
| In The Light | Power moves with Sarah Jakes Roberts | Seen Head Known Loved | let (self) love rule | [https://podcasts.apple.com/us/podcast/in-the-light-with-toni-tone/id1551222888](https://podcasts.apple.com/us/podcast/toni-told-me-with-toni-tone/id1787009373) |
| ADHD Experts | 502 psychedelic therapy for mood disorders | 501 ADHD & Procrastivity | 500 identifying depression and anxiety in teens with ADHD | <https://podcasts.apple.com/us/podcast/adhd-experts-podcast/id302139296> |
| That Conversation | E16 Whole lotta SEX-healing body trauma, sexual agency, growing beyond judgement | E15 BOTTLED UP: anger, pain, sadness | E14: Voices: is that me? Anxiety? Or god? ) | <https://podcasts.apple.com/us/podcast/that-conversation/id1594268869> |
| Being Well | Understanding the "flight" response: anxiety, avoidance, and feeling safe, | managing the fight response: anger, repression, and self-regulation | everything you need to know about therapy | <https://podcasts.apple.com/us/podcast/being-well-with-forrest-hanson-and-dr-rick-hanson/id1120885936> |
| The Squeeze | Tyler Cameron: Or what? | Amy Loughren: The real good nurse | Mayim Bialik: life is in session | [https://podcasts.apple.com/us/podcast/the-squeeze/id1665487022](https://podcasts.apple.com/us/podcast/the-squeeze/id1665203594) |
| Bible At Bedtime | Revelation 4 & Psalms 119 | Courage to Conquer 2 | Devotion proverbs 1 | <https://podcasts.apple.com/us/podcast/bible-at-bedtime/id1490921543> |
| Boring Book For Bedtime | The principles of Chemistry, by Dmitri Mendeleev, part 5 | The railway traveler’s handy book, part 2 | 1890 Gardiner and Co. Seed Annual 1: Flower Novelties | <https://podcasts.apple.com/us/podcast/boring-books-for-bedtime/id1437487865> |
| Black Girl Burnout | living whole heartedly with Kelsey Ellis-part 1 | Grown Woman Talk with Dr. Sharon Malone-part 3 | Grown Woman talk with Dr. Sharon Malone-part 2 | <https://podcasts.apple.com/us/podcast/black-girl-burnout/id1507014700> |
| Cleaning up your mental mess | introducing the kevin rose show | Navigating the fertility | how guilt can make us better people | <https://podcasts.apple.com/us/podcast/cleaning-up-your-mental-mess-with-dr-caroline-leaf/id1334767397> |
| This Jungian Life | identity crisis: when our story falls apart | six swans Jungian analysis: transforming fear into joy | encounters with light: jung’s near-death experiences | <https://podcasts.apple.com/us/podcast/this-jungian-life-podcast/id1376929139> |
| Just Sleep | the Gelston slander by Herbert Jenkins | the marvelous land of oz: the prisoners of the queen | the jellyfish and the monkey-Japanese fairy tales | <https://podcasts.apple.com/us/podcast/just-sleep-bedtime-stories/id1439383258> |
| This Naked Mind | EP 700: Reader’s Question-how do I beat alcohol cravings? | EP 699: Naked life story-Mary F. | EP698: Reader’s question-what’s the link between alcohol and depression. | <https://podcasts.apple.com/us/podcast/this-naked-mind-podcast/id1210690432> |
| Oversharing | baby steps: navigating the emotional and financial toll of recurrent pregnancy loss Ft. Lori Vogt Rosone | Am I annoying for always playing devil’s advocate? | | <https://podcasts.apple.com/us/podcast/oversharing-with-kelsey-and-taryn/id1661073773> |
| The Best of You | episode 104: overcoming the fear of vulnerability | Episode 103: name frame, and brave gossip | episode 102: I shouldn’t feel conflicted about God | <https://podcasts.apple.com/us/podcast/the-best-of-you/id1640524936> |
| Sober Cast | workshop: ego and self-bob A (part1 of 5) | Esh: Jenine M, | Panel meeting: we are not the future | <https://podcasts.apple.com/us/podcast/sober-cast/id1452238087> |
| I Have ADHD | 264 feeding the mind: debunking the diet-only ADHD management myth | 263. everything on my mind lately no.2 | 262 how coaching can change your life with Brooke Schmidtian | <https://podcasts.apple.com/us/podcast/i-have-adhd-podcast/id1442681753> |
| Better Tomorrow | Find your color (w/Hannah Garber | opposites attract (w/Becca Tilley & Tanya Rad) | Time Heals All (w/Paul Bassagoitia) | [https://podcasts.apple.com/us/podcast/better-tomorrow/id1516289882](https://podcasts.apple.com/us/podcast/better-tomorrow-with-hannah-brown/id1694610238) |
| Listen to Sleep | the Hedgehogs’ twilight quest | ease your mind-a guide meditation to calm racing thoughts | the misty mountains’ embrace | <https://podcasts.apple.com/us/podcast/listen-to-sleep/id1603781218> |
| Daily Mindfulness with Calm | calm conversations: and then we rise | plan for contingencies, | stories | <https://podcasts.apple.com/us/podcast/daily-mindfulness-with-calm/id1599545558> |
| Guided Meditation | body love sleep meditation | mental health break sleep meditation | panic attack relief meditation | <https://podcasts.apple.com/us/podcast/guided-meditation/id1467012731> |
| The Chalene Show | a weekend full of mayhem, destruction and death | the better body, brain and sex hormone | slow your scroll | <https://podcasts.apple.com/us/podcast/the-chalene-show/id839421827> |
| Black Girls Heal | #245: the first 3 steps of healing love avoidance | #244: 4 ways family trauma will normalize emotional abuse | #243: 3 ways love addicts sabotage their relationships | <https://podcasts.apple.com/us/podcast/black-girls-heal/id1456781684> |
| Psychology Unplugged | gaslighting: how to respond | conduct disorder | parental triggers | <https://podcasts.apple.com/us/podcast/psychology-unplugged/id1484693024> |
| The Heare Brotherhood | Nathan Tanner from panic attack to triathlons | Jimmy Rex starting we are the they | Josh Prows discovering a creative career | <https://podcasts.apple.com/us/podcast/the-heare-brotherhood/id1528553088> |
| Radio Headspace | Embracing chaos with Andy Puddicombe | practicing solitude | how prioritizing changed my life with Andy Puddicombe | [https://podcasts.apple.com/us/podcast/radio-headspace/id1435681236](https://podcasts.apple.com/us/podcast/radio-headspace/id1510981488) |
| Sleep Cove | Intense sleep hypnosis | Alice through the looking glass | for sleep-guided meditation for deep serene sleep | <https://podcasts.apple.com/us/podcast/sleep-cove-sleep-hypnosis/id1237218112> |
| I Can’t Sleep | Cicada | Mario | Curling | <https://podcasts.apple.com/us/podcast/i-cant-sleep/id1478498048> |
| The Morning Ritual | Dana Slamp on what it means to recognize the divine within | Meditation: caring for your whole self | merging spirituality & business with kara ladd | <https://podcasts.apple.com/us/podcast/the-morning-ritual/id1481382372> |
| Hacking Your ADHD | ADHD and pregnancy with dusty chipura | Finding your groove with joshua greene | crafting your ADHD survival kit with maddy de Gabriele | <https://podcasts.apple.com/us/podcast/hacking-your-adhd/id1460809989> |
| Do you F*cking Mind? | 337 Encore: how to be self-assured and comfortable in your own skin, | 336 painful in laws and how to deal with them | 334. how to really stop caring about what people think of you | <https://podcasts.apple.com/us/podcast/do-you-f-cking-mind/id1455295468> |
| Girl Just Heal | oh, she gon’ bounce back! | the journey is personal | a way out of no way | <https://podcasts.apple.com/us/podcast/girl-just-heal/id1549208462> |
| SelfHealers Soundboard | episode 99 how to create authentic friendships | episode 98 how to forgive yourself | episode 97 how to create safety in your body | <https://podcasts.apple.com/us/podcast/selfhealers-soundboard/id1485596673> |
| Drift Off | the sleep tale of Samuel Whiskers | the relaxing bedtime story of Maida’s little house chapter | Japanese folktales for sleep: the tea kettle & the good thunder | <https://podcasts.apple.com/us/podcast/drift-off/id1533488016> |
| Sober Motivation | Garrett struggled for years attempting to moderate his alcohol intake | Jen’s journey began with teenage drinking to fit in | For saz moderating her alcohol intake never worked | <https://podcasts.apple.com/us/podcast/sober-motivation/id1545879741> |
| Just Break Up | Your hurt deserves to be seen | Undetectable is Untransmittable | Is this a red flag or a real boundary? | <https://podcasts.apple.com/us/podcast/just-break-up/id1571234726> |
| Neuro Diversity Podcast | OCD: Beyond the Neat & Orderly | Autism + ADHD = AuDHD | Experiential Therapy: Less Talk & More action | <https://podcasts.apple.com/us/podcast/the-neurodiversity-podcast/id1476171740> |
| The Badass Counseling show | Q&A: Narc Boyfriend, dysfunctional relationship, and more | pt2: American Hero Slays Demons! | Q&A: In-LAws, Narc Dads, Abusive Parents, and More | <https://podcasts.apple.com/us/podcast/the-badass-counseling-show/id1481174101> |
| Boundaries.me | Episode 411 The Dr. Cloud show-the upside of struggle | The Dr. Cloud Show-Mastering the effective apology | the Dr. Cloud show-rekindling the marital flame | <https://podcasts.apple.com/us/podcast/boundaries-me-with-dr-john-townsend/id1452777919> |
| Wake Me Up | morning self worth & self love Affirmations | self love & self worth meditation feel good about yourself! | positive affirmations for success, wealth, and | <https://podcasts.apple.com/us/podcast/wake-me-up/id1488948985> |
| Daily Meditation Podcast | Elemental Mastery: A Samurai’s Meditation, Day 6: the circle of 5 rings mediation series | Root Chakra Grounding: the samurai’s foundation, day 5: the circle of 5 rings meditation series | Mudra of Balance: elemental harmony, day 4 the circle of 5 rings meditation series | <https://podcasts.apple.com/us/podcast/daily-meditation-podcast/id1099004981> |
| The Anxiety Coaches | Reducing anxiety by quieting the noise | classic ACP 627 7 easy practices to quiet your anxiety | classic ACP to avoid anxious overthinking | <https://podcasts.apple.com/us/podcast/the-anxiety-coaches-podcast/id771681509> |
| MindValley Show | how to get unstuck: 7 habits to break the cycle | how to bounce back from pain and use it for your greatest growth | maria conceicao: from stewardess to record-breaker on a mission to help children in poverty | <https://podcasts.apple.com/us/podcast/mindvalley-podcast/id1437858457> |
| Recovery Elevator | RE 484: Recovery is a Sandwich | RE 483: NA Beers | RE 482: Advice for the newly sober | <https://podcasts.apple.com/us/podcast/recovery-elevator/id1041907600> |
| Dopey | Dopey 478: How David Riggins Burned off his leg while Super high on Fentanyl and found recovery and other dumb sh!t | Dopey Tuesday Teaser! Alan and Robbie! | Dopey 477: Hunting Serial Killers Operating under the cloak of America’s opioid epidemic | <https://podcasts.apple.com/us/podcast/dopey-on-the-dark-comedy-of-addiction/id1192428261> |
| Being Known | S9E10: Full Circle | S9E9: Hope | S9E8: Character | <https://podcasts.apple.com/us/podcast/being-known/id1476779266> |
| The Vault | Sahaj Kohli: What To Do when your family doesn’t approve of therapy, | Benita Alexander: learning to trust yourself again after trauma | Erin McGoff: How to talk about Mental health in the Workplace | <https://podcasts.apple.com/us/podcast/the-vault-with-tianna-and-jade/id1620501498> |
| Down To Sleep | Pride and Prejudice Audio book (part 3) | | The tales of beatrix potter | <https://podcasts.apple.com/us/podcast/down-to-sleep/id1584456773> |
| Sober Powered | E224: Alcohol makes your brain stop working | E223: 6 common triggers for drinking and how to avoid them | E222: 6 Neurotransmitters that get messed up by alcohol | <https://podcasts.apple.com/us/podcast/sober-powered/id1519983487> |
| Sober Mom Life | SobrieTEA: the vanderpunp rules reunion & the end of Carl and Lindsay | A sobriety update with Julia Dzafic of Lemon Stripes, | The real sober moms with Karen | <https://podcasts.apple.com/us/podcast/sober-mom-life/id1590148042> |
| Letting it Settle | Guided Meditation: Coming home | Mini Meditation: Coming home | How I learned to come home to myself | <https://podcasts.apple.com/us/podcast/letting-it-settle/id1537836283> |
| Carlat Psychiatry | Contingency Management with Dr. Dominick Dephilippis | Wounded Healers Impairment & Strength, | Throwback The bright Side of the Serotonin Gene | <https://podcasts.apple.com/us/podcast/carlat-psychiatry-podcast/id1378638085> |
| Think Positive | Think positive: Daily affirmation June 9 | Think positive: Daily affirmation June 8 | Think positive: Daily affirmation June 7 | <https://podcasts.apple.com/us/podcast/think-positive-daily-affirmations/id1451601771> |
| Ghost of a Podcast | 436: Horoscope-squares to Saturn & Pluto + Deeply Mercurial Vibes | 435: Guilt to Gratitude | 434: Horoscope-a very busy week with a very busy new moon in Gemini | <https://podcasts.apple.com/us/podcast/ghost-of-a-podcast/id1448695447> |
| Mental Illness Happy Hour | #699 Harnessing Anxiety-Dr Arash Javanbakht | #698 ADHD in Marriage-Penn&Kim Holderness | #697 Best of Chris Fairbanks-growing up around dementia | <https://podcasts.apple.com/us/podcast/mental-illness-happy-hour/id427133154> |
| Daily Affirmations for Woman | I am going to be okay | I release the burden of overwhelm | I have unshakeable faith in the perfect outcome in every area of my life | <https://podcasts.apple.com/us/podcast/daily-affirmations-for-women/id1489673738> |
| 21 Days of Abundance | Day 22: happiness and humanity (so hum) | Day21: Living abundantly (so hum) | day20: Living luxury (Om Ridham Namah | <https://podcasts.apple.com/us/podcast/21-days-of-abundance/id1511918374> |
| Uniquely Human | Supporting siblings of children and family members with disabilities with Emily boll | About "Ezra" with Director Tony Goldwyn, writer Tony Spiridakis and Casting Consultant | De-pathologizing series EP 4-Autism: A blessing?? A tragedy?? With Barry and Dave | <https://podcasts.apple.com/us/podcast/uniquely-human-the-podcast/id1539313560> |
| The Narcissist’s Code | why is it so hard for narcissists to admit when they are wrong? | why so many narcissists think that they are good people regardless of how they are treating you | when a narcissists asks you for "Space" | <https://podcasts.apple.com/us/podcast/the-narcissists-code/id1490754696> |
| Well Now | The isolation of a life-threatening diagnosis | How doulas Make childbirth safer for everyone | "as little regulation as guns": how social media hurts youth mental health | <https://podcasts.apple.com/us/podcast/well-now/id1479579918> |
| Back From the Borderline | childhood emotional neglect part 11: healing (transforming suffering into strength and crafting a new narrative | childhood emotional neglect part 10: healing (reclaiming your power and understanding threat responses | exposing miss USA’s dark secrets: title holder resignations, misogyny, corruption, and everywhere like such as | <https://podcasts.apple.com/us/podcast/back-from-the-borderline/id1581005460> |
| Relax with Animal Facts | Ocelots: Shy But Deadly Kitties | Humpback Whales: Singing Giants | Atlas Moth | <https://podcasts.apple.com/us/podcast/relax-with-animal-facts/id1515854835> |
| The Anxiety Dr. | #140 Childhood Trauma and Anxiety | #139 Vitamin and Minerals that May Help Your Anxiety | Foods that May Ease our Anxiety | <https://podcasts.apple.com/us/podcast/the-anxiety-dr-podcast/id1498673424> |
| Meditation For Woman | Oh So Tired | Sleep Sounds: Concluding an Eventful Day | My heart is Full | <https://podcasts.apple.com/us/podcast/meditation-for-women/id1499834874> |
| The Recovery Show | Finding Strength in Mutual Aid: Insights from AI-Anon’s Third Tradition-409 | A deep Dive into Sharing in Meetings-408 | Spencer T talk at 71st Greeley AA Stampede | <https://podcasts.apple.com/us/podcast/the-recovery-show/id721379615> |
| Waking up to Narcissism | DARVO, Differentiation and Detangling Difficult Dynamics | When life just “Lifes” All over you: A tale of resilience and growth through Narcissistic relationships | Death By a Thousand Cuts VII-The Rise of the emotionally mature! | <https://podcasts.apple.com/us/podcast/waking-up-to-narcissism/id1551050891> |
| The ADHD Women’s Wellbeing podcast | Are you ready to make small shifts to create big changes? | the spiritual technology and wisdom to transform lives | the top 5 supplements to take for ADHD | https://podcasts.apple.com/us/podcast/adhd-womens-wellbeing-podcast/id1605386171 |
| Feeling Good | The Deep Freeze, Part 2 (of 2), | The Deep Freeze, Part 1(of 2), | Ask David: Assertiveness, suppressing your feelings, the “Miracle Cure” question | <https://podcasts.apple.com/us/podcast/feeling-good-podcast-team-cbt-the-new-mood-therapy/id1240159641> |
| Mentally Stronger | 115-Finding Calm in the Present Moment with Michael Galyon | 114-Break Free From a Bad Mood by Identifying the Mood Boosters that will help you feel better fast | 113-overcoming the fear of feeling with author Jennie Allen | <https://podcasts.apple.com/us/podcast/mentally-stronger-with-therapist-amy-morin/id1437549512> |
| The Mindset Babe | 160 Mindset series: and it’s okay!!! | 159 thoughts holding you back! | 158. why your manifestation hasn’t happened yet!!! | <https://podcasts.apple.com/us/podcast/the-mindset-babe-podcast/id1495047445> |
| Hello Someday | 10 Sober Travel Tips you need for your next alcohol-free vacation | why emotional sobriety is key to navigating an alcohol-free life | how to rekindle romance and manage conflict in your relationship | <https://podcasts.apple.com/us/podcast/hello-someday-podcast/id1484596882> |
| Meditation Minis | From Negative Feelings to Pure flow | Evening "cleaning" meditation | Backyard chill vibes meditation | <https://podcasts.apple.com/us/podcast/meditation-minis-podcast/id1124795486> |
| Real Pod | "I had a baby via surrogate!" Mallory Goldman’s motherhood journey, autoimmune disease, &supportive hubby | The beach is not about your body: pep talk with Vic | My journey with therapy (Why I go when I started, & my best advice) | <https://podcasts.apple.com/us/podcast/real-pod/id1469685724> |
| Surviving Narcissism | Evan Rachel Wood on surviving her abusive relationship with Marilyn Manson | Ask Dr. Ramani: How to go "no contact" a Narcissist | My famous Parents are Narcissists with Juliet Landau | <https://podcasts.apple.com/us/podcast/surviving-narcissism/id1521440691> |
| 2 Sober Girls | 89: Morning Routines + Starting your day with purpose, | 88: our evening routines | 87: navigating peer pressure + staying sober in social situations | <https://podcasts.apple.com/us/podcast/2-sober-girls/id1530827745> |
| The Gabby Reece Show | #271 GUT expert: these are the seven fiber rich foods you need | #279 mindset expert: she went from the battlefield in Iraq to congress w/ Tulsi Gabbard | #269 He’s a Top competition surfer after suffering brain damage | <https://podcasts.apple.com/us/podcast/the-gabby-reece-show/id1486716048> |
| Emotionally Uncomfortable | 1133: [Attracting profit]"Finding purpose: Maximizing time and becoming your best self" | 1132: [parenting] "Depression & Creativity in Kids & Adults" | 1131: [Attracting profit] "the subtle ways women sabotage time management (and it has nothing to do with work or kids)" | <https://podcasts.apple.com/us/podcast/emotionally-uncomfortable/id1541582716> |
| Counselor Toolbox podcast with DocSnipes | 20 ways attitude impacts your physical and mental health and 6 tips to improve it | 8 ways childhood trauma and adverse childhood experiences impact (ACEs) Adult health and behavior) | 5 Activities to develop emotional intelligence and maturity | <https://podcasts.apple.com/us/podcast/counselor-toolbox-podcast-with-docsnipes/id991655548> |
| Soul Sistas Sleep Meditation - Guide for Black Women | Meditation for endless thoughts guided sleep meditation | what do we need? a national women’s history month meditation | black history month celebration meditation a celebration of US! | <https://podcasts.apple.com/us/podcast/soul-sistas-sleep-meditation-guide-for-black-women/id1562249225> |
